# Epidemiology of injury in rural Pondicherry, India

**DOI:** 10.5249/jivr.v3i2.74

**Published:** 2011-07

**Authors:** Ganapathy Kalaiselvana, Amol R. Dongre, T. Mahalakshmy

**Affiliations:** ^*a*^Department of Community Medicine, Sri Manakula Vinayagar Medical College, Pondicherry, India.

## Abstract

**Background:**

To find out the prevalence of "all" injuries, its nature, outcome and sources of treatment among rural population of Pondicherry.

**Methods:**

It was a triangulated study of quantitative (survey) and qualitative (Focus Group Discussion, FGD) methods. The trained second year medical undergraduate students paid house visits to all houses in five feasibly selected villages of our field practice area. The students interviewed the housewife and obtained information for all injuries for each family member in last one year and its sources of treatment. We could obtain information for 1,613 (96.7%) households. Post-survey, FGDs were undertaken to explore the various traditional treatments for the common injuries. The data was entered and analyzed using Epi_info 6.04d software package.

**Results:**

Overall, the prevalence of all injury among all age groups was 30.6% in last one year. Injuries were significantly more after 18 years of age and among men (p was less than 0.001). About 99.2% injuries reported were accidental and majority (58.2%) went to government doctor for treatment. Most common causes of injuries were fall on the ground from height or due to slip (7.4%), road traffic accidents (5.6%), agriculture related injuries (5%) and bites by scorpion/insects/snakes /dogs (4.1%). FGDs explored some potentially harmful traditional remedial measures at village level such as application of mud or cow dung on the injury and burning the site of thorn prick on foot sole.

**Conclusions:**

Considering the high prevalence of all injuries related to road traffic accidents, fall from height and agriculture work related injuries across all age groups, especially among men and some potentially harmful traditional treatments, an intervention in the form of targeted injury prevention program for different age and sex group, focusing health education efforts based on local epidemiology and behavioral practices is needed.

## Introduction

Injuries are becoming major public health problem worldwide. According to recent estimates, each year over 5 million people around the world die as a result of an injury.^1^In India, injury ranks next only to diarrhoeal diseases and respiratory infections. It is estimated that by 2020, the injury would be the prime contributor in the total disease burden.^2^National Sample Survey Organization of India found that the poor households have to spend a high amount for treatment of severe injuries than other diseases.^2^Injuries disproportionately affect the poor in low and middle income countries.^3^As India is passing through a major socio-demographic, epidemiological, and technological transition; injuries are coming up as emerging public health problem.^4^Injury by definition means that there is a body lesion due to an external cause, either intentional or unintentional, resulting from a sudden exposure to energy (mechanical, electrical, thermal, chemical or radiant) generated by agent–host interaction.^1^

Injuries are no longer perceived as random, unavoidable events but rather as ones that are largely preventable. In order to develop effective prevention strategies, community based epidemiological information on the burden of injuries is required to develop and implement mechanisms for prevention and control of injuries. Community-based injury surveys have one overriding advantage over hospital-based surveillance methods in that they capture injuries that fail to reach hospitals, i.e. those injury deaths occurring in the community, injuries that are treated outside the formal health sector and minor injuries that do not necessarily require hospital attention.^1^There are very few population based studies to understand the various causes of injuries in predominant rural population of India.^3^Hence, the objective of the present rural community based study was to determine know the prevalence of all injuries, its nature, outcome and sources of treatment in a rural population.

## Methods

The present triangulated study of quantitative (survey) and qualitative (Focus Group Discussion, FGD)^5^method was undertaken in the villages of a Primary Health Centre (PHC) in rural Pondicherry. Out of seven villages of a PHC area having total population of 13,837, five villages having total population of 8,920 (64.5%) were feasibly selected as these villages are our field practice villages of Rural Health Training centre. Pondicherry (renamed as Puducherry), a union territory of India, having population of 9,73829  is located on the Malabar Coast, at 162 kms south of Chennai, the capital of Tamil Nadu. It has tropical climate, 81.5% literacy and 45% people have agriculture as a main occupation.^6^

To begin with, social mapping was done in all five villages. There were 1, 668 households in study villages. The trained second year medical undergraduate students paid home visit to all houses during October - November 2009, which are relatively free, pre-monsoon months for the villagers. We used National Family Health Survey – III interviewer’s manual for sensitizing the students on survey techniques.^7^ A trained team of 30 medical students were taken to the villages in morning hours to interview housewife assuming that females are the most likely to know the injury history of all household members.^1^After obtaining informed consent, information on injuries for each of the family members in last one year and its sources of treatment was collected from the responding housewife using pre-designed and pre-tested questionnaire. For the purposes of the study, as in the case of the injury surveillance guidelines, the term “injury” was used to describe the physical damage that results when a human body is suddenly or briefly subjected to intolerable levels of energy. It can be a bodily lesion resulting from acute exposure to energy in amounts that exceed the threshold of physiological tolerance, or it can be an impairment of function resulting from a lack of one or more vital elements (i.e. water, air, warmth), as in drowning, strangulation or freezing. All injuries within the recall period of one year were considered. Disability was defined as a temporary loss of function for more than one week or permanent loss of function (partial or complete) of part of the body.^1^The information on injury was collected and classified according to ‘mechanism of injury’ such as road traffic accidents, fall, poisoning etc. The recall was aided by a structured checklist of common injuries. Students were also encouraged to explore the various traditional remedies for injuries and note it at the end of the questionnaires. To enhance the quality of the data, completed questionnaires were checked for the completeness of the information on the same day of the field survey and feedback was ensured before next day’s survey. Five percent of the questionnaires were re-checked by the team of four social workers who supervised the survey. We could obtain information for 1,613 (96.7%) households, 20 (1.2%) houses were locked and remaining 35 (2.1%) refused to participate in the study. We referred to guidelines for conducting community surveys on injuries and violence given by World Health Organization for planning this study.^1^

Post-survey, FGDs were undertaken to explore the various home remedies for injuries and their rationale behind such practices. Two FGDs were conducted with men and women each i.e. total four FGDs were conducted. The FGDs were facilitated by the social worker in local language Tamil using structured guidelines and note taker recorded it. There were 8-12 purposively selected participants in each meeting, who were willing to participate and talk freely. FGD participants were selected from different socio-economic strata. A trained local social worker indentified the participants, obtained their consent and scheduled the meeting at the day and time convenient to them. FGD meetings were conducted at village Gram-panchayat (building for local self-government) where all members would not hesitate to come. The duration for each was approximately one hour. Facilitators arranged refreshments for the participants at the end of meeting.

The quantitative data was entered and percentages and odds ratios were calculated with 95% Confidence Intervals (CI) using Epi_info 6.04d software package. The level of significance was fixed at p < 0.05. The qualitative information obtained from students’ exploration of traditional treatment during survey, field notes of social workers and FGD data was transcribed in English, manually coded and analyzed over a period of two months.^8^A list of priori codes on ‘common mechanism of injuries’ was used to code the text data. The content analysis was manually done. The unit of the analysis was statements under a given code. Nine codes were clubbed under four broad categories. FGD findings are presented as non-hierarchical typology of common injuries and its local level management.^9^These findings were reviewed by two internal faculties in the department of Community Medicine, who were working in the local area for the past three years.

Ethical principles such as obtaining consent from the respondents and ensuring confidentiality were adhered to, throughout the study. Ethical clearance was obtained from the ethics committee of the Institute.

## Results

Overall, the prevalence of all injury in the study population was 30.6%. Among injured, 561 (22.2%) were below the age of 18 years, 1886 (34.2%) were adults (18-60 years) and 213 (33.1%) were older individuals (60+years) (p<0.001). The prevalence of injuries among male was significantly higher 1432 (33.3%) than in women 1228 (28.0%) (P<0.001). About 99.2% of reported injuries were accidental.  Majority i.e. 1547 (58.2%) of injured persons were taken to the government doctor for treatment, 499 (18.8%) to private doctor, 398 (15%) used home remedies, 204 (7.7%) went to the village-level faith healers and only 12 (0.4%) went to the village Anganwadi workers (AWW) or to Auxiliary Nurse Midwife (ANM). Most of the injuries, 2382 (89.5%) were reported to be recovered, 264 (9.9%) led to disability and remaining 14 (0.5%) resulted in death.

Table 1, has given percent distribution of mechanism of injuries across different age groups and sex with odds ratio. Most common injuries which occurred among individuals < 18 years old, were falls from height/due to slip [183 (7.2%)], bites by scorpion/snake/dogs [129 (5.1%)], and burns [70 (2.8%)]. In adults (18-60 years), common injuries were bites by scorpion/snake/dogs [463 (8.4%)], falls from height/ due to slip [394 (7.1%)], road traffic accidents [388 (7.0%)] and agricultural injuries [337 (6.2%)]. However, among older population (60+ years), 63 (9.8%) had history for fall on ground from height/ due to slip and 58 (9%) had bites by scorpion/snake/dogs. Among male, prevalence was high for road traffic accidents (9.1%) and fall on the ground from height (8.1%). Among female, prevalence was high for bites by scorpion/insect etc (8.1%) and fall on ground from height (6.7%). Overall, odds ratio for all injuries was significantly high (OR=1.52; 95%CI-1.36-1.70) in age group 18-60 years and in males (OR=1.52; 95%CI-1.36-1.70) (P<0.05). Among adults (18-60 years), odds ratio for road traffic accidents, agricultural injury and bits by scorpion/insects etc. were significantly higher with reference to below 18 years. Among old age (60+ years), the odds ratio for fall on the ground from height was significantly higher with reference to below 18 years (OR=1.39; 95%CI-1.02-1.89) (p<0.05). Except for injuries like burns, bites and insecticide poisoning, the odds ratio for all other injuries was significantly higher in males than females (p<0.05).

**Table:** Table 1:**Distribution of mechanism of injuries according to age group and sex in rural Pondicherry**

Injury in last one year	Age groups			Sex		Total n=8682
	Under 18 n= 2523	18-60 n=5516	60+ n=643	Female n=4382	Male n=4300	
Road traffic accidents n(%)	68(2.7)	388(7.0)	33(5.1)	97(2.2)	392(9.1)	489(5.6)
OR (95%CI)	1	2.73(2.09-3.58)*	1.95(1.25-3.05)*	1	4.43(3.51-5.59)*	
Fall on the ground from height/ due to slip n(%)	183(7.2)	394(7.1)	63(9.8)	293(6.7)	347(8.1)	640(7.4)
OR (95%CI)	1	0.98(0.82-1.18)	1.39(1.02-1.89)*	1	1.23(1.04-1.44)*	
Injury during agricultural work, n(%)	59(2.4)	337(6.2)	33(5.1)	195(4.4)	234(5.4)	429(5.0)
OR (95%CI)	1	2.72(2.04-3.64)*	2.26(1.43-3.56)*	1	1.24(1.01-1.51)*	
Burns (Fire, lightening, electric shock), n(%)	70(2.8)	188(3.4)	15(2.3)	188(4.3)	85(2.2)	273(3.1)
OR (95%CI)	1	1.24(0.93-1.65)	0.84(0.46-1.51)	1	0.45(0.34-0.59)*	
Insecticide Poisoning, n(%)	14(0.6)	34(0.6)	3(0.5)	33(0.7)	19(0.4)	51(0.6)
OR (95%CI)	1	1.11(0.58-2.18)	0.84(0.19-3.12)	1	0.58(0.32-1.06)	
Bites by scorpion/insects/snakes/dogs etc, n(%)	129(5.1)	463(8.4)	58(9.0)	356(8.1)	294(6.8)	650 (4.1)
OR (95%CI)	1	1.70(1.38-2.09)*	1.84(1.32-2.57)*	1	0.83(0.70-0.98)*	
Drowning, n(%)	4(0.2)	8(0.1)	2(0.3)	4(0.09)	10(0.2)	14 (0.2)
OR (95%CI)	1	0.91(0.25-3.60)	1.96(0.25-12.41)	1	2.55(0.74-9.62)*	
Others	34(1.3)	74(1.3)	6(0.9)	62(1.4)	52(1.2)	114(1.3)
Total	561(22.2)	1886(34.2)	213(33.1)	1228(28.0)	1432(33.3)	2660(30.64)
OR (95%CI)	1	1.52(1.36-1.70)*	1.73(1.43-2.10)	1	1.52(1.36-1.70)*	

*p <0.05

As per the respondents of FGD, the broad categories of common injuries that emerged from FGD data were agriculture injuries, bites, burns and poisoning. We could also explore the various village-level remedies practiced for injured person before consulting the medical doctor which is summarized inTable 2. Local remedies ranged from use of locally available herbs, coconut, salt, tamarind, and turmeric to potentially harmful practices such as application of fine dust, cow dung, sickle rust on wound and burning the site of thorn prick on sole with the match stick fire. For snake bite and scorpion bite village-level faith healer (natvaidya) is consulted who gives herbal and sanctified thread. When there is no relief with village-level treatment or in presence of pus formation in the injured wound, then the person is taken to the government health care facility (Table 2).

**Table:** Table 2:**Summary of FGDs: Common injuries and local treatment for injuries**

Category	Codes	Local treatment
Agriculture injuries	Cut injury by farm weapons	Juice of local herb called ‘karsalankanni’ is applied on wound. Some apply fine dust on the wound and cover it with wet cloth. The practice of applying mixture of ash of white cloth and sugar or turmeric or coconut oil on wound was reported. The mixture of lime and sugar or simply lime or sickle rust is often used for dressing the cut wound.
	Injury by domestic animals	Injured part of the body is rubbed against the animal head or face of the animal. This is said to help better healing.
	Thorn pricks	The practice such as application of white milk of the local herb; putting match stick tip powder at the site and burn it was reported.
	Blunt injuries	Mixture of tamarind paste and salt is applied over the site. Some apply turmeric paste. It is said to reduce swelling.
Bites	Snake bite	A thread is tied above the wound; cuts around the bite mark are made to suck out the blood; and patient is given banana bark juice to drink. Use of local herb called ‘Sriyanangai’ and ‘Perianangai’ is common practice. The victim is asked to consume black hen’s blood and green leaves together to neutralize the poison.
	Scorpion bite	Use of local herb called ‘Sriyanangai’ and ‘Perianangai’ is common. Faith healer is consulted for a sanctified thread. Kerosene is applied over site for cooling effect. The person is given coconut or coconut water to eat or drink. Some people seek herbal treatment from village-level faith healer called natvaidya.
	Dog bite	Cow dung is applied over the bite site and warm oil is applied. The victim is given little warm oil orally to remove dog’s poison. Pachalli leaves (Erraukam) are boiled with coconut oil which is then applied over the wound and part of it is given to drink to the injured person.
Burns	Burn due to fire	Juice of banana stem or crushed potato is applied on the site, Use of turmeric, butter, salt, ink, kerosene, mixture of milk and oil is also reported.
	Electric shock	A plenty of water is advised to drink and tomato is given to eat.
Poisoning	Insecticide poisoning	A mixture of cow dung and tamarind is given to swallow to induce vomiting.

## Discussion

Overall, the prevalence of all injury among all age groups was 30.6% in last one year. A study in rural Haryana reported 8% prevalence of agriculture related injuries.10 In the present study, the overall prevalence of agriculture related injuries was 5% which was (6.2%) in 18-60 years of age group. The road traffic injuries were high among males and above 18 years of age. Various studies on injuries were conducted in India, Sri Lanka, Uganda and Nigeria.^11,12,13,14^But, the findings of these studies could not be compared with the present study due to differences in sampling, operational definitions of injury, type of respondents (self vs. proxy) and type of data analysis.

Injuries were significantly more prevalence for those 18 years and older and among men. About 99.2% of the reported injuries were accidental and majority (58.2%) went to government doctor for treatment. In India, it was found that injured patients relied more on free health services of public sector institutions.^2^FGDs explored some potentially harmful remedial measures being practiced at village level such as applying mud or cow dung on the injury and burning the site of thorn prick on foot sole. Varghese in rural Haryana also explored some traditional treatment for agricultural injuries such as urinating on wound as first aid, application of oils and fats on burns and bruises, application of heated paste of flour, vegeta-bles, turmeric, leaves and other materials.^10^

It is noteworthy that almost all reported injuries were accidental in nature, which could have been prevented by care and protective measures. Unfortunately, injuries are still thought to be due to fate.^3^Majority of the injured persons went to or were taken to government doctor (58.2%), but utilization of village-level services of Anganwadi workers (AWW) and Auxiliary Nurse Midwife (ANM) was found to be less than one percent. Fifteen percent of the injured people received traditional treatments, some of which were found potentially harmful. First aid measures for injuries in the farms were found inadequate and inappropriate. It has been found that traditional methods are commonly used for agricultural injuries and some of these were found to prolong the healing time of injuries well beyond the expected.^10^Findings of this study warrant need for community-based health education projects focusing on pre-hospital care, life skill development and behavior change among rural Indian population. Apart from health education efforts, there is a need to strengthen government supported village-level health care facilities.

The present community-based   triangulated   research describes the local situation of injuries in different age groups and explores the various potentially harmful village-level remedies, which can be useful for development of injury surveillance and prevention. Borse et al. recommended that developing countries like India and China should take initiative in not only reducing the burden of unintentional injuries by appropriate research but also publishing such work and contribute to the global pool of knowledge.^15^The qualitative data pointing to the use of potentially harmful remedies at village-level can be used for development of locally relevant health education material. Since we followed the World Health Organization guidelines for injury survey, future studies with similar guidelines can use the present findings for comparison. The limitations of the present study should be kept in mind. It was a small scale study based on a non-probability sample. One of the most significant limitations of community-based surveys is rooted in their reliance on self-reporting by respondents. The accuracy of respondents’ answers on the occurrence of injury events or the duration of the resultant disability cannot be independently verified. The residence housewife was taken as a proxy respondent for all other members with the assumption that she would better recall injuries for all the other family members.

Considering the high prevalence of injuries related to road traffic accidents, fall from height and agricultural-related injuries across all age groups, especially among men, as well as evidence of using potentially harmful traditional treatments, injury prevention interventions are needed to target different age and sex group. These interventions should learn from the local epidemiology and focus on changing injury-related risky behaviors and practices. In addition to the health education efforts, there is a need to strengthen village-level health care facilities to provide appropriate first-aid treatments for different injuries.

## References

[1] Sethi D, Habibula S, McGee K, Peden M. Guidelines for conducting community surveys on injuries and violence. Int j inj contr saf prom 2004, http://www.informaworld.com/index/713817814.pdf, accessed 3 March 2010. 10.1080/156609704/233/327505 15903167

[2] Gumber A. Cost and Burden of Injury in India: An Emerging Public Health Threat. http://www.iussp.org/Brazil2001/s70/S74_03_Gumber.pdf, accessed 5 April 2010.

[3] World heath organization. Child injury prevention in the South-East Asia region.Geneva: WHO,2008.

[4] Gururaj G. Injuries in India: A national perspective. In: Burden of Disease in India: Equitable development - Healthy future. New Delhi: National Commission on Macroeconomics and Health. Ministry of health and family welfare, Government of India, 2005.

[5] Dawson S, Manderson L, Tallo VL. The focus group manual: Methods for social research in disease. Boston: International Nutrition Foundation for Developing Countries (INFDC), 1993.

[6] Pondicherry location. http://pondicherryonline.in/Profile/Geography/, accessed 3 March 2010.

[7] National Family Health Survey-III -India. Interviewer’s manual 2006, http://www.nfhsindia.org/manuals.html, accessed 3 March 2010.

[8] Hsieh HF, Shannon SE (2005). Three approaches to qualitative content analysis. Qual Health Res.

[9] Donald R. 15 methods of data analysis in qualitative research, http://qualitativeresearch.ratcliffs.net/15methods.pdf, accessed 12 December 2008.

[10] Varghese M, Mohan D (1990). Occupational injuries among agricultural workers in rural Haryana, India. Journal of Occupational Accidents.

[11] Garg N, Hyder AA (2006). Road traffic injuries in India: a review of the literature. Scand J Public Health.

[12] Lamawansa M, Piyathilake A (2008). Incidence of physical injuries in rural community in Sri Lanka: result of the first community survey in Sri Lanka. Indian J Community Med.

[13] Kobusingye O, Guwatudde D, Lett R (2001). Injury patterns in rural and urban Uganda. Inj Prev.

[14] Olawale OA, Owoajie ET (2007). Incidence and pattern of injuries among residents of a rural area in South-Western Nigeria: a community-based study. BMC Public Health.

[15] Borse NN, Hyder AA (2009). Call for more research on injury from the developing world: results of a bibliometric analysis. Indian J Med Res.

